# A risk signature with four autophagy‐related genes for predicting survival of glioblastoma multiforme

**DOI:** 10.1111/jcmm.14938

**Published:** 2020-02-17

**Authors:** Yulin Wang, Weijiang Zhao, Zhe Xiao, Gefei Guan, Xin Liu, Minghua Zhuang

**Affiliations:** ^1^ Department of Neurosurgery The First Affiliated Hospital of Shantou University Medical College Shantou China; ^2^ Wuxi School of Medicine Jiangnan University Wuxi China; ^3^ Department of Neurosurgery The First Hospital of China Medical University Shenyang China; ^4^ Department of Stomatology The First Affiliated Hospital of Shantou University Medical College Shantou China

**Keywords:** autophagy, glioblastoma multiform, nomogram, prognosis, risk signature

## Abstract

Glioblastoma multiforme (GBM) is a devastating brain tumour without effective treatment. Recent studies have shown that autophagy is a promising therapeutic strategy for GBM. Therefore, it is necessary to identify novel biomarkers associated with autophagy in GBM. In this study, we downloaded autophagy‐related genes from Human Autophagy Database (HADb) and Gene Set Enrichment Analysis (GSEA) website. Least absolute shrinkage and selection operator (LASSO) regression and multivariate Cox regression analysis were performed to identify genes for constructing a risk signature. A nomogram was developed by integrating the risk signature with clinicopathological factors. Time‐dependent receiver operating characteristic (ROC) curve and calibration plot were used to evaluate the efficiency of the prognostic model. Finally, four autophagy‐related genes (DIRAS3, LGALS8, MAPK8 and STAM) were identified and were used for constructing a risk signature, which proved to be an independent risk factor for GBM patients. Furthermore, a nomogram was developed based on the risk signature and clinicopathological factors (IDH1 status, age and history of radiotherapy or chemotherapy). ROC curve and calibration plot suggested the nomogram could accurately predict 1‐, 3‐ and 5‐year survival rate of GBM patients. For function analysis, the risk signature was associated with apoptosis, necrosis, immunity, inflammation response and MAPK signalling pathway. In conclusion, the risk signature with 4 autophagy‐related genes could serve as an independent prognostic factor for GBM patients. Moreover, we developed a nomogram based on the risk signature and clinical traits which was validated to perform better for predicting 1‐, 3‐ and 5‐year survival rate of GBM.

## 1 INTRODUCTION

Glioblastoma multiforme (GBM) is one of the most aggressive types in glioma with 5‐year survival rate of 5%.^1^ Although current treatment approaches, including maximum safe resection and adjuvant chemoradiotherapy, have been adopted, the median overall survival is only about 15 months.^2^ Despite the progress of experimental technologies and therapeutic regimens in this field, such as inhibition of oncogenic signal transduction, anti‐angiogenesis and immunotherapy, GBM remains incurable.^3^ It is thus necessary to explore novel biomarkers or targets for GBM treatment.

Recent study has showed that autophagy is involved in tumorigenesis and development of GBM.^4^ Autophagy is a cellular self‐digestive process that protects cells via eliminating damaged or abandoned intracellular components under the conditions of hypoxia, oxidative stress or nutrient starvation.^5^ Autophagy can govern the fate of cancer cells via initiating pro‐survival or pro‐death mechanisms.^6^ As a first‐line chemotherapeutic agent, temozolomide (TMZ) has shown benefit for prolonging the survival of GBM patients.^7^ TMZ preferentially induces autophagic death in GBM cells rather than apoptosis through PI3K/AKT/mTOR signalling pathway.^8^ However, further study demonstrates that persistent inhibition of PI3K/AKT/mTOR by TMZ can only induce autophagy transiently and promote drug resistance of GBM.^9^, ^10^ In the meantime, combination with autophagy inhibitors or regulators can interfere with the therapeutic effects of TMZ for GBM.^11^ These researches reveal that autophagy‐targeted therapy is a promising approach to potentiate the efficacy of conventional therapies in GBM.

In this study, we identified four autophagy‐related genes associated with the prognosis of GBM patients from the TCGA, REMBRANDT and Gravendeel data sets. A risk signature was established based on the four genes and proved to be an independent risk factor for GBM patients. Furthermore, we developed a nomogram that integrated the risk signature with clinicopathological factors (IDH1 status, age and experience of radiotherapy or chemotherapy) and validated its better performance for predicting 1‐, 3‐ and 5‐year survival rate of GBM patients.

## 2 MATERIALS AND METHODS

### 2.1 Data source

Autophagy‐related genes were extracted from Human Autophagy Database (HADb, http://www.autophagy.lu/index.html) and the GO_AUTOPHAGY gene set in Gene Set Enrichment Analysis website (http://software.broadinstitute.org/gsea/index.jsp). The two gene sets were combined and integrated into an autophagy‐related gene set. Gene expression data, clinical characteristics and survival information in The Cancer Genome Atlas (TCGA, HG‐UG133A microarray and GBMLGG RNA‐seq), Repository for Molecular Brain Neoplasia Data (REMBRANDT, microarray) and Gravendeel data sets (microarray) were downloaded from GlioVis (http://gliovis.bioinfo.cnio.es/).^12^ In the GlioVis online data set, RNA‐seq data processing is based on the normalized count reads from the pre‐processed data (sequence alignment and transcript abundance estimation) with log2 transformation after adding a 0.5 pseudocount. For microarray data, the “affy” package was used for robust multi‐array average normalization followed by quantile normalization.

### 2.2 Construction of a risk signature associated with survival of GBM patients

To screen genes for constructing risk signature, univariate Cox regression models were performed to select genes that are associated with overall survival of GBM patients in the TCGA (HG‐UG133A platform), REMBRANDT and Gravendeel data sets. *P* < .05 was considered statistical significance. Overlapping autophagy‐related genes were extracted from the three data sets and visualized in a venn diagram. Least absolute shrinkage and selection operator (LASSO) regression was used to screen out the optimal gene combination for constructing the risk signature. Multivariate Cox regression model was carried out to further identify the selected genes using “step” function in R language. The data in the TCGA database (HG‐UG133A) were used as the training cohort, and data in the Gravendeel and REMBRANDT data sets were used for the validation cohorts. Subsequently, a risk signature was established based on a linear combination of the regression coefficient derived from the multivariate Cox regression model coefficients and expression level of the genes. The risk score formula was calculated as follows: Risk score = (expr_gene1_ × Coef_gene1_) + (expr_gene2_ × Coef_gene2_) + … + (expr_genen_ × Coef_genen_).^13^ The GBM patients were classified into low‐risk group and high‐risk group according to the median value of the risk scores. The Kaplan‐Meier (K‐M) method and time‐dependent receiver operating characteristic (ROC) curve were used to assess the efficiency of the risk signature.

### 2.3 Establishment and assessment of the nomogram

For better clinical application of the risk signature, patients in the TCGA data set (HG‐UG133A) with detailed information about age, IDH status and experience of radiotherapy or chemotherapy were included. Univariate and multivariate Cox regression analyses were performed to determine the association between these factors (risk score, age, IDH status and history of radiotherapy or chemotherapy) and patients' overall survival. The included patients were divided into training cohort (60%) and validation cohort (40%) randomly using R package “caret.” The training cohort was used to establish a nomogram for predicting 1‐, 3‐ and 5‐year survival rate of GBM patients via R programming language. The validation cohort was used for internal validation. ROC curve and calibration plot were carried out to evaluate the efficiency of the nomogram.

### 2.4 Functional enrichment analysis

Gene set enrichment analysis (GSEA, https://www.broadinstitute.org/gsea/index.jsp) was performed to identify the autophagy‐related gene sets between LGG and GBM.^14^ Normalized enrichment score (NES) and false discovery rate (FDR) were applied to determine the statistical differences. The gene set variation analysis (GSVA) was used to explore biological processes and Kyoto Encyclopedia of Genes and Genomes (KEGG) pathways associated with the risk signature.^15^ Gene sets with differences between high‐risk group and low‐risk group in TCGA data set (GBM HG‐UG133A) were selected using the R package “limma,” and adjust *P* value < .05 was considered statistically significant. Several representative gene sets were presented in heatmaps. To confirm the KEGG pathways associated with the signature, R package “clusterProfiler” was performed on the differentially expressed genes (DEGs) between low‐risk group and high‐risk group which were selected via “limma” package in R with adjust *P* value < .05 and |log2(fold change)| > 0.5.^16^ The KEGG pathway map was presented by “pathview” package.

### 2.5 Statistical analyses

All the statistical analyses including principal component analysis (PCA), univariate and multivariate Cox regression models, LASSO regression, ROC curve analysis and K‐M survival analyses were performed using Rstudio (version 3.5.2). Quantitative data were exhibited as the mean ± standard deviation (SD). Statistical differences were compared by Wilcoxon test between two groups and Kruskal‐Wallis H for multigroup comparison. *P* < .05 was considered statistically significant. The venn, heatmaps, boxplots, pie charts, forest plots and calibration plots were drawn using R language.

## 3 RESULTS

### 3.1 Four autophagy‐related genes were screened out for constructing a risk signature

**Figure 1:**
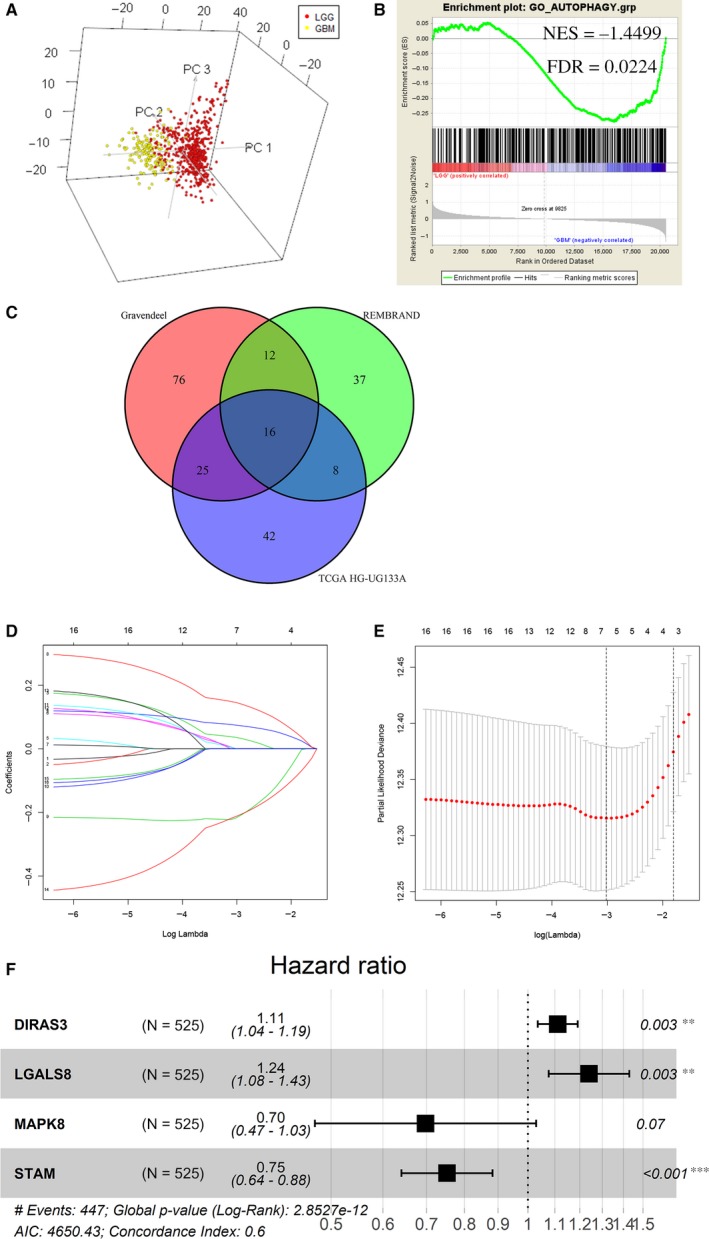
Screening out genes for constructing a risk signature. A, Principal components analysis (PCA) of autophagy‐related genes between LGG and GBM. B, Gene set enrichment analysis (GSEA) for comparing autophagy gene term between LGG and GBM. C, Overlapping genes associated with GBM survival in the TCGA, REMBRANDT and Gravendeel database. D, Log (Lambda) value of the 16 genes in LASSO model. E, The most proper log (Lambda) value in LASSO model. F, Four genes (DIRAS3, LGALS8, MAPK8 and STAM) were selected for constructing a risk signature using multivariate Cox regression model

A total of 531 autophagy‐related genes were integrated from HADb database and the GO_AUTOPHAGY gene set in GSEA website (Table S1). PCA based on these autophagy‐related genes confirmed the distribution difference between low‐grade glioma (LGG) and glioblastoma multiforme (GBM) in the TCGA (GBMLGG RNA‐seq) data set. As shown in Figure 1A, GBM samples were located on the left side, and LGG samples were on the other side. To identify autophagy‐related biological processes between LGG and GBM, GSEA was performed and the results showed that autophagy‐related genes were highly enriched in GBM (Figure 1B), suggesting that autophagy played an essential role in GBM. Our study just focused on GBM based on these results of PCA and GSEA. Ninety‐one genes in the TCGA HG‐UG133A, 73 genes in the REMBRANDT and 129 genes in the Gravendeel data set were found to be correlated with GBM survival using univariate Cox regression analysis (Table S2, *P* < .05). Sixteen overlapping genes in the three databases were screened out and visualized in a venn diagram (Figure 1C). LASSO regression analysis was performed on the overlapping genes so as to avoid overfitting problems in risk signature, and 7 genes (CTSB, DIRAS3, HK2, LGALS8, MAPK8, PPP1R15A and STAM) were retained according to the optimal lambda value (Figure 1D,E, log(lambda.min) = −3.1114). Multivariate Cox regression analysis was adopted to further identify an appropriate gene combination for establishing the risk signature using “step” function in R software. Finally, 4 genes (DIRAS3, LGALS8, MAPK8 and STAM) were selected (Figure 1F). Among the four genes, DIRAS3 and LGALS8 were risk factors for GBM survival with HR > 1, and MAPK8 and STAM were protective factors with HR < 1. Consistent with the results, K‐M survival curves showed patients with higher expression levels of MAPK8 or STAM had favourable outcomes (Figure S1A,B, *P* < .05) and patients with higher expression levels of DIRAS3 or LGALS8 had poor prognosis in GBM (Figure S1C,D, *P* < .05).

### 3.2 Establishment of a risk signature with four autophagy‐related genes

**Figure 2:**
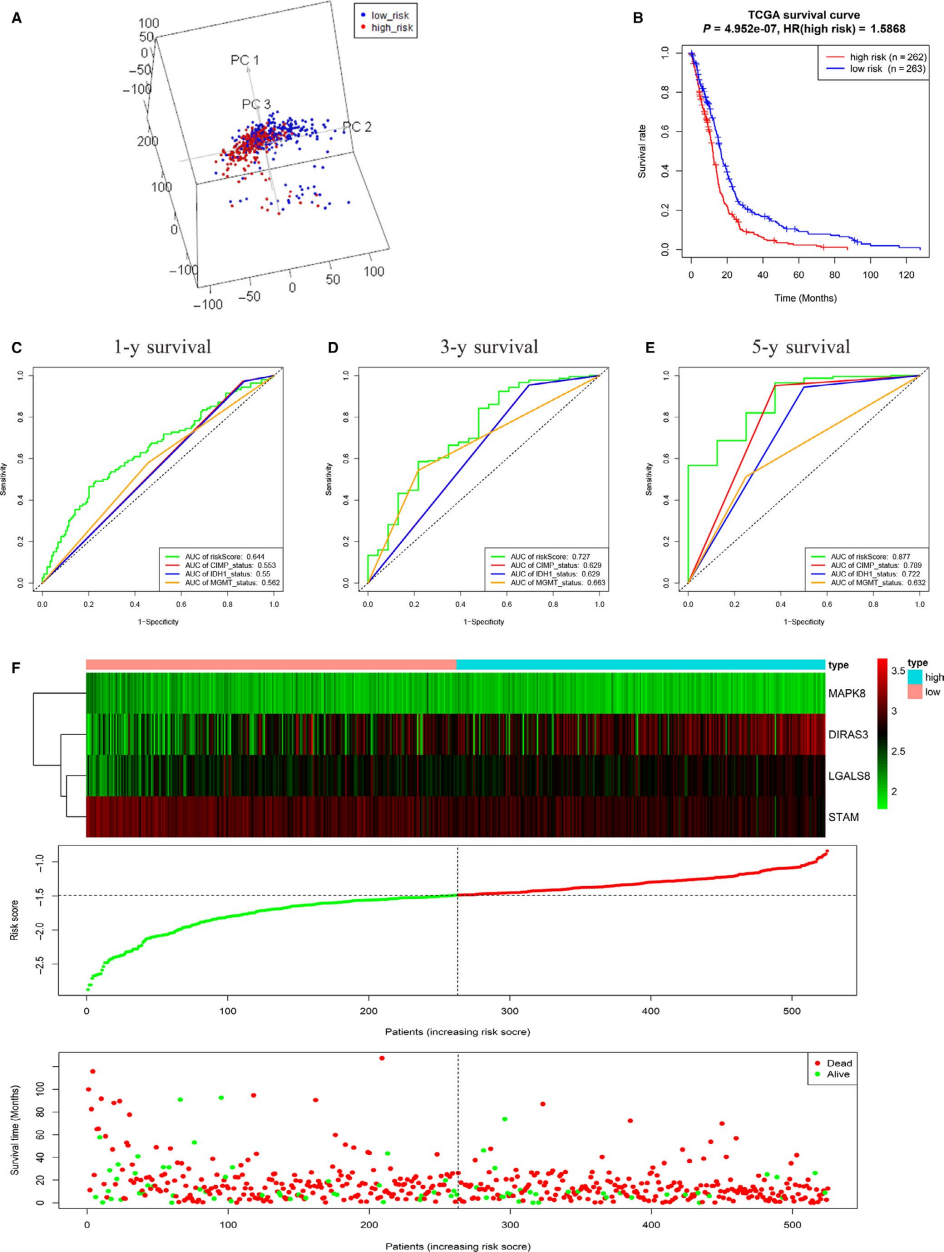
Establishment of the risk signature with four autophagy‐related genes in the TCGA database. A, PCA based on genome expression data between low‐risk group (n = 263) and high‐risk group (n = 262). B, Kaplan‐Meier survival curves showed the prognostic value of the risk signature between low‐risk group (n = 263) and high‐risk group (n = 262). C‐E, ROC curves were used to assess the efficiency of the risk signature for predicting 1‐ (C), 3‐ (D) and 5‐y survival (E). F, The four genes expression profiles, the risk scores distribution and patients' survival status in the TCGA database

A total of 525 GBM patients in TCGA HG‐UG133A platform were used to establish a risk signature. As mentioned in the method, the risk signature was constructed based on the expression levels of the four genes and the regression coefficient derived from the multivariate Cox regression model. The risk score for each patient was calculated as follows: risk score = (0.1052 × expression level of DIRAS3) + (0.2152 × expression level of LGALS8) + (−0.3603 × expression level of MAPK8) + (−0.2851 × expression level of STAM). The patients were divided into high‐risk and low‐risk groups according to the median cut‐off value of the scores. To explore the difference between low‐risk and high‐risk groups, PCA was implemented based on genome expression data and the results demonstrated the distribution difference between the two groups (Figure 2A). In addition, patients in the high‐risk group had significantly worse overall survival than those in the low‐risk group (Figure 2B, *P* < .0001). Considering the key roles of IDH1, MGMT and G‐CIMP in GBM,^17^, ^18^ ROC curves were used to compare the efficiencies of the risk signature with these biomarkers in prognostic prediction. As shown in the ROC curves, the area under curves (AUCs) of the risk signature for predicting the 1‐, 3‐ and 5‐year survival were 0.644 (Figure 2C), 0.727 (Figure 2D) and 0.877 (Figure 2E), respectively, which were larger than those of IDH1, MGMT promoter and G‐CIMP status. With the increase in the risk scores, the expression levels of MAPK8 and STAM were decreased and the expression levels of DIRAS3 and LGALS8 were up‐regulated (Figure 2F). In the meantime, the number of alive patients reduced (Figure 2F).

### 3.3 Validation of the risk signature

**Figure 3:**
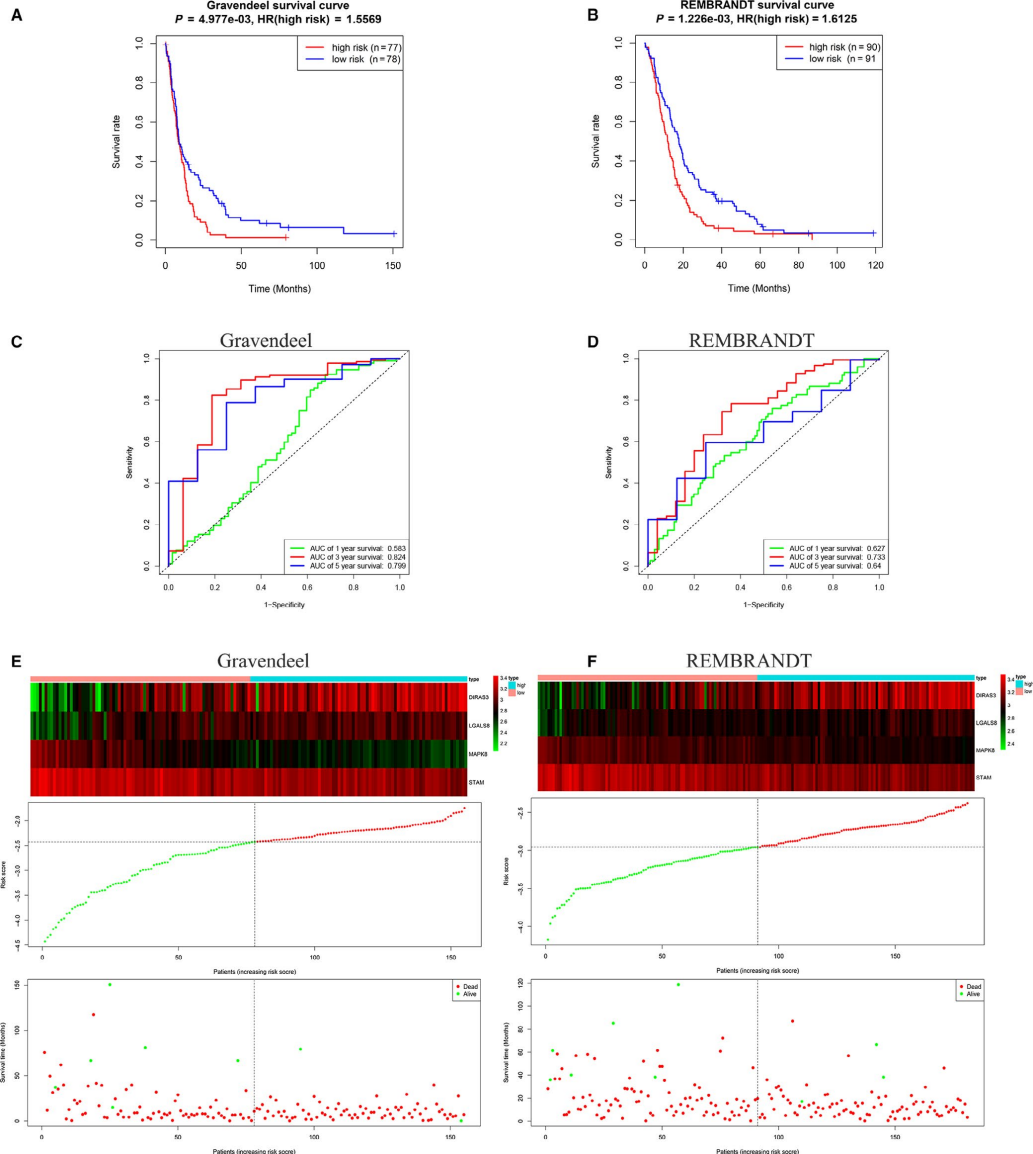
Evaluating the efficiencies of the risk signature in the REMBRANDT and Gravendeel data sets. A, B, Kaplan‐Meier survival curves showed the prognostic value of the risk signature in Gravendeel data set (A. low‐risk group, n = 78; high‐risk group, n = 77; *P* < .01) and REMBRANDT database (B. low‐risk group, n = 91; high‐risk group, n = 90; *P* < .01). C, D, ROC curves evaluated the efficiency of the risk signature for predicting 1‐, 3‐ and 5‐y survival in Gravendeel data set (C) and REMBRANDT database (D). E, F, The four genes expression profiles, the risk scores distribution and patients' survival status in the Gravendeel data set (E) and REMBRANDT database (F)

A total of 155 GBM samples in the Gravendeel data set and 181 GBM samples in the REMBRANDT database were collected and used for two validation data sets to assess the performance of the risk signature. The K‐M survival curves showed that patients with higher risk scores had poorer prognosis than those with lower risk scores (Figure 3A, Gravendeel, *P* < .01 and B, REMBRANDT, *P* < .01). The AUCs of ROC curves for predicting 1‐, 3‐ and 5‐year survival of GBM in the Gravendeel data set were 0.583, 0.824 and 0.799, respectively (Figure 3C), and those in the REMBRANDT database were 0.627, 0.733 and 0.64, respectively (Figure 3D). Consistent with the results in the TCGA, with the increase in the risk scores, the expression levels of MAPK8 and STAM were down‐regulated and the expression levels of DIRAS3 and LGALS8 were up‐regulated both in the Gravendeel (Figure 3E) and in the REMBRANDT (Figure 3F) database. Concomitantly, the overall survival and the number of alive patients declined (Figure 3E,F). These results indicated the risk signature performed well for predicting the survival of GBM patients.

### 3.4 Association between the risk signature and clinical characteristics

**Figure 4:**
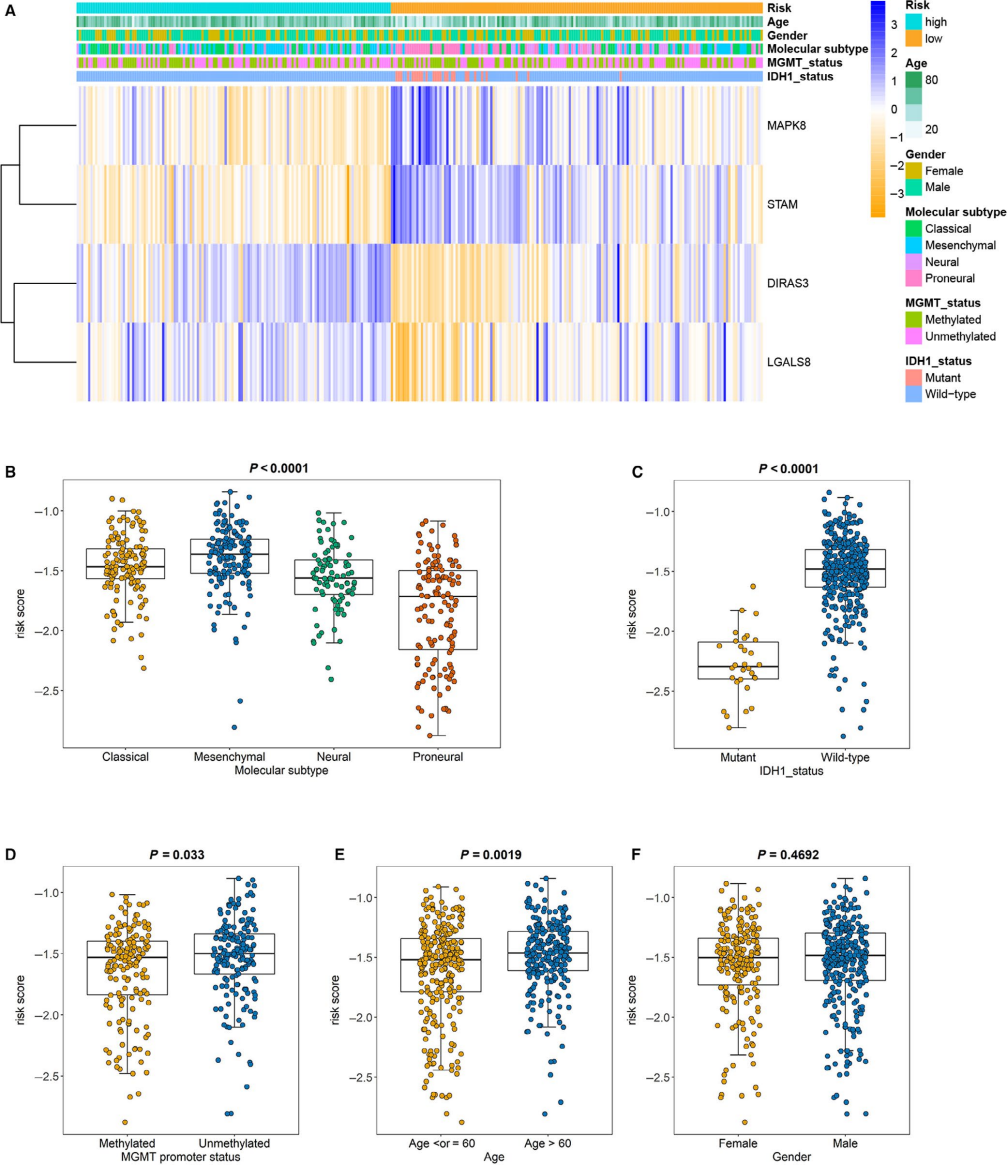
Associations between the signature risk scores and clinical features. A, The heatmap showed the associations between the risk signature and the clinical characteristics (age, gender, molecular subtypes, MGMT promoter status, IDH1 status and survival status) in the TCGA database. B‐F, Distribution of the risk scores in different cohorts stratified by the molecular subtype (B, classical, n = 144; mesenchymal, n = 156; neural, n = 88; proneural, n = 137; *P* < .0001), IDH1 status (C, mutant, n = 30; wild‐type, n = 372; *P* < .0001), MGMT promoter status (D, methylated, n = 170; unmethylated, n = 176; *P* = .033), age (E, age > 60, n = 248; age ≤ 60, n = 271; *P* = .002) and gender (F, female, n = 203; male, n = 314; *P* = .469)

To explore the association between the risk signature and clinical characteristics, we firstly developed a heatmap to present the distribution trends of age, gender, molecular subtypes, MGMT promoter status and IDH1 status between low‐risk and high‐risk groups in the TCGA database. As shown in Figure 4A, high‐risk group inclined to contain more elder patients, whereas samples with IDH1 mutant were all in low‐risk group. To be more intuitive, pie charts were used to display the proportion of each factor (age, gender, molecular subtypes, MGMT promoter status and IDH1 status) in low‐risk and high‐risk groups. Similar results were shown in Figure S2A, elder patients and samples with mesenchymal subtype or classical subtype accounted for a large proportion in the high‐risk group. And there were no significant differences between low‐risk and high‐risk groups for gender and MGMT promoter status. Subsequently, we compared the risk scores in different cohorts stratified by molecular subtypes, age, IDH status, MGMT promoter status and gender separately. The risk scores in the mesenchymal subtype were obviously higher than those in neural and proneural subtypes (Figure 4B, *P* < .0001). For IDH status, the risk scores decreased in patients with IDH1 mutant type compared with IDH1 wild‐type (Figure 4C, *P* < .0001). The risk scores of patients with MGMT promoter methylation were lower than MGMT promoter unmethylation (Figure 4D, *P* = .033). Patients above 60 years old inclined to have higher risk scores compared with those in the younger age group (Figure 4E, *P* = .002). However, there was no difference in risk scores between male and female (Figure 4F, *P* = .468).

**Figure 5:**
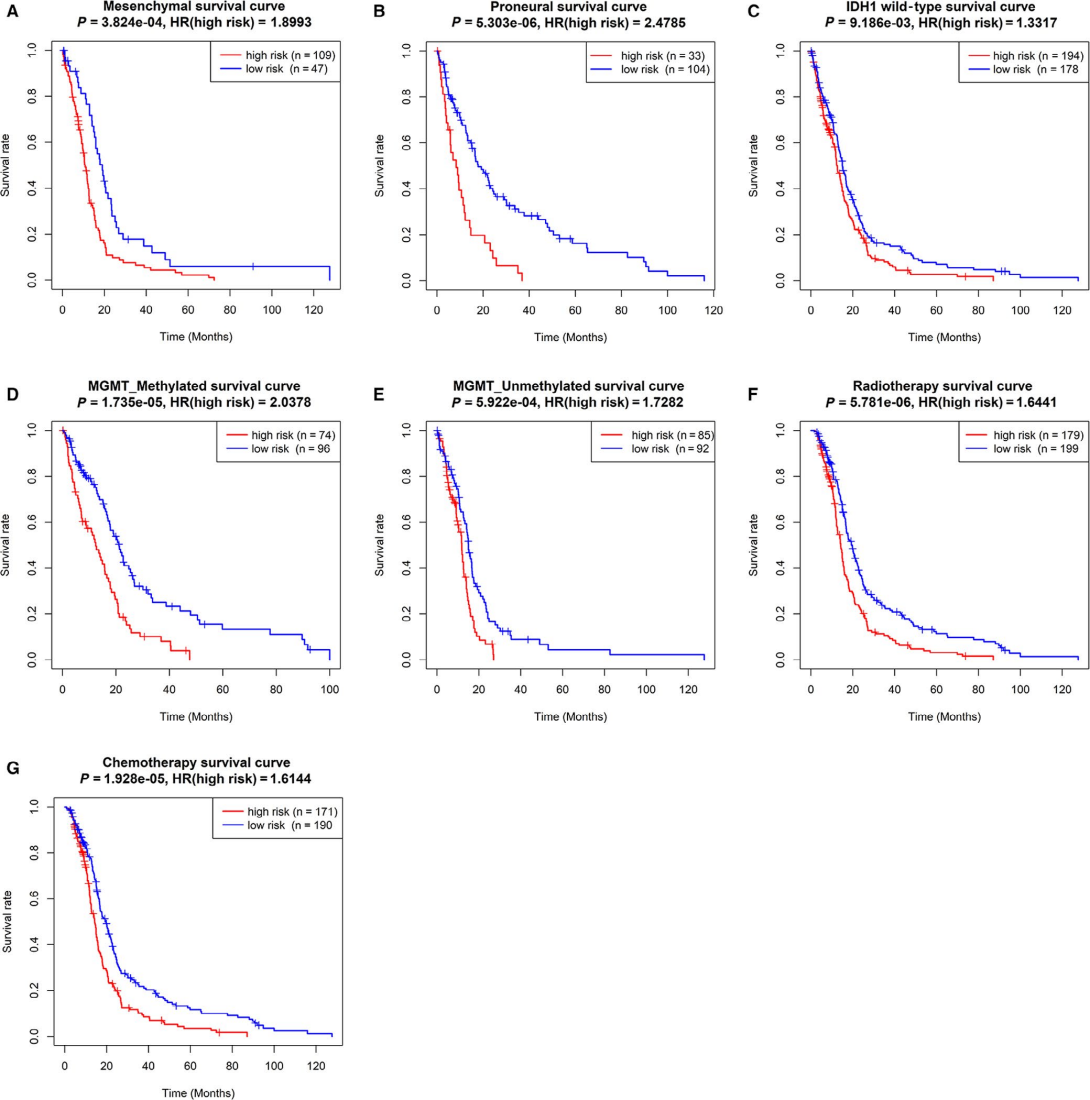
Kaplan‐Meier survival curves showed prognostic values of the risk signature in different cohorts. Prognostic values of the risk signature in different cohorts stratified by mesenchymal and proneural subtypes (A‐B), IDH1 wild‐type (C), MGMT promoter status (D‐E) and history of radiotherapy (F) or chemotherapy (G)

Afterwards, we also explored the prognostic value of the risk signature in different cohorts stratified by molecular subtypes, MGMT promoter status, IDH1 status and history of radiotherapy or chemotherapy. In the four different molecular subtypes, higher risk scores indicated poor prognosis in the mesenchymal (Figure 5A, *P* < .001) and proneural (Figure 5B, *P* < .0001) subtypes, but there were no prognostic differences between low‐risk group and high‐risk group in the classical (Figure S3A, *P* = .9447) and neural (Figure S3B, *P* = .5887) subtypes. The patients in high‐risk groups had adverse outcomes in the IDH1 wild‐type group (Figure 5C, *P* < .01), MGMT promoter methylation group (Figure 5D, *P* < .0001) and MGMT promoter unmethylation group (Figure 5E, *P* < .001). Furthermore, higher risk scores were also found to be clinically associated with poor prognosis in patients receiving radiotherapy (Figure 5F, *P* < .0001) or chemotherapy (Figure 5G, *P* < .0001).

### 3.5 Construction of a nomogram for predicting 1‐, 3‐ and 5‐year survival rate of GBM

**Figure 6:**
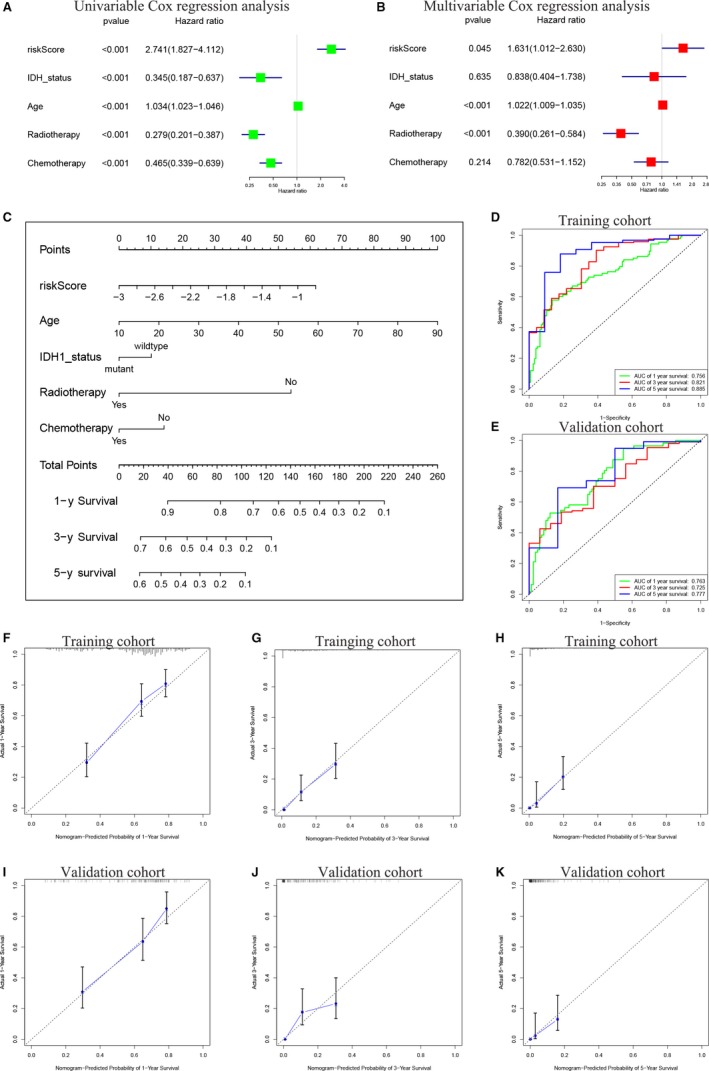
Construction of a nomogram for predicting 1‐, 3‐ and 5‐y survival of GBM. A, B, Univariate and multivariate Cox regression analyses evaluated the contribution of each variable to GBM survival in the training cohort. C, A nomogram for predicting 1‐, 3‐ and 5‐y survival rate of GBM patients was established. D, E, ROC curves evaluated the efficiency of the nomogram for predicting 1‐, 3‐ and 5‐y survival in the training cohort (D) and validation cohort (E). F‐H, Calibration curves showed the probability of 1‐ (F), 3‐ (G) and 5‐y survival (H) between the prediction and the observation in the training cohort. I‐K, The calibration curves for predicting patients' survival at 1‐ (I), 3‐ (J) and 5‐y survival (K) in the validation cohort

In order to better apply the risk signature, we collected 401 GBM patients in the TCGA HG‐UG133A platform with detailed clinical information including IDH1 status, age and history of radiotherapy or chemotherapy. The 401 samples were randomly divided into a training cohort (n = 241) and a validation cohort (n = 160) (Table 1). The training cohort was used for constructing a nomogram to predict survival of GBM, and the validation cohort was used for further assessing the efficiency of the nomogram. Firstly, we performed univariate and multivariate Cox regression analyses in the training cohort, indicating that the risk signature was an independent risk factor for GBM patients (Figure 6A,B, *P* < .05). Subsequently, a nomogram integrating the five factors was constructed for predicting 1‐, 3‐ and 5‐year survival rate of GBM. In the nomogram, the patients' 1‐, 3‐ and 5‐year survival rates were estimated by the total points obtained by adding up the point of each factor (Figure 6C). ROC curve and calibration plot were applied to evaluate the performance of the nomogram. The AUCs of ROC curves for predicting 1‐, 3‐ and 5‐year survival were 0.756, 0.821 and 0.885, respectively, in the training cohort (Figure 6D) and 0.763, 0.725 and 0.777, respectively, in the validation cohort (Figure 6E). The calibration curves showed good agreements between the prediction and observation in the training cohort (Figure 6F‐H) and in the validation cohort (Figure 6I‐K) for the probabilities of 1‐, 3‐ and 5‐year survival. These results indicated that the nomogram demonstrated good accuracy for predicting 1‐, 3‐ and 5‐year survival rates of GBM patients.

**Table 1:** Demographics and clinicopathologic characteristics of patients in training cohort and validation cohort for construction and evaluation of the nomogram

Character	Training cohort (n = 241)		Validation cohort (n = 160)	
	NO. of patients	%	NO. of patients	%
Risk score				
Median	−1.5709		−1.5603	
Range	−2.8081 to −0.8840		−2.8778 to −0.8396	
Age, y				
Median	58.5		59.5	
Range	19 to 89.3		20.4 to 88.6	
IDH status				
Mutant	19	7.8838	10	6.2500
Wild‐type	222	92.1162	150	93.7500
Radiotherapy				
Yes	178	73.8589	115	71.8750
No	63	26.1411	45	28.1250
Chemotherapy				
Yes	176	73.0290	114	71.2500
No	65	26.9710	46	28.7500

### 3.6 Functional analysis of the risk signature

**Figure 7:**
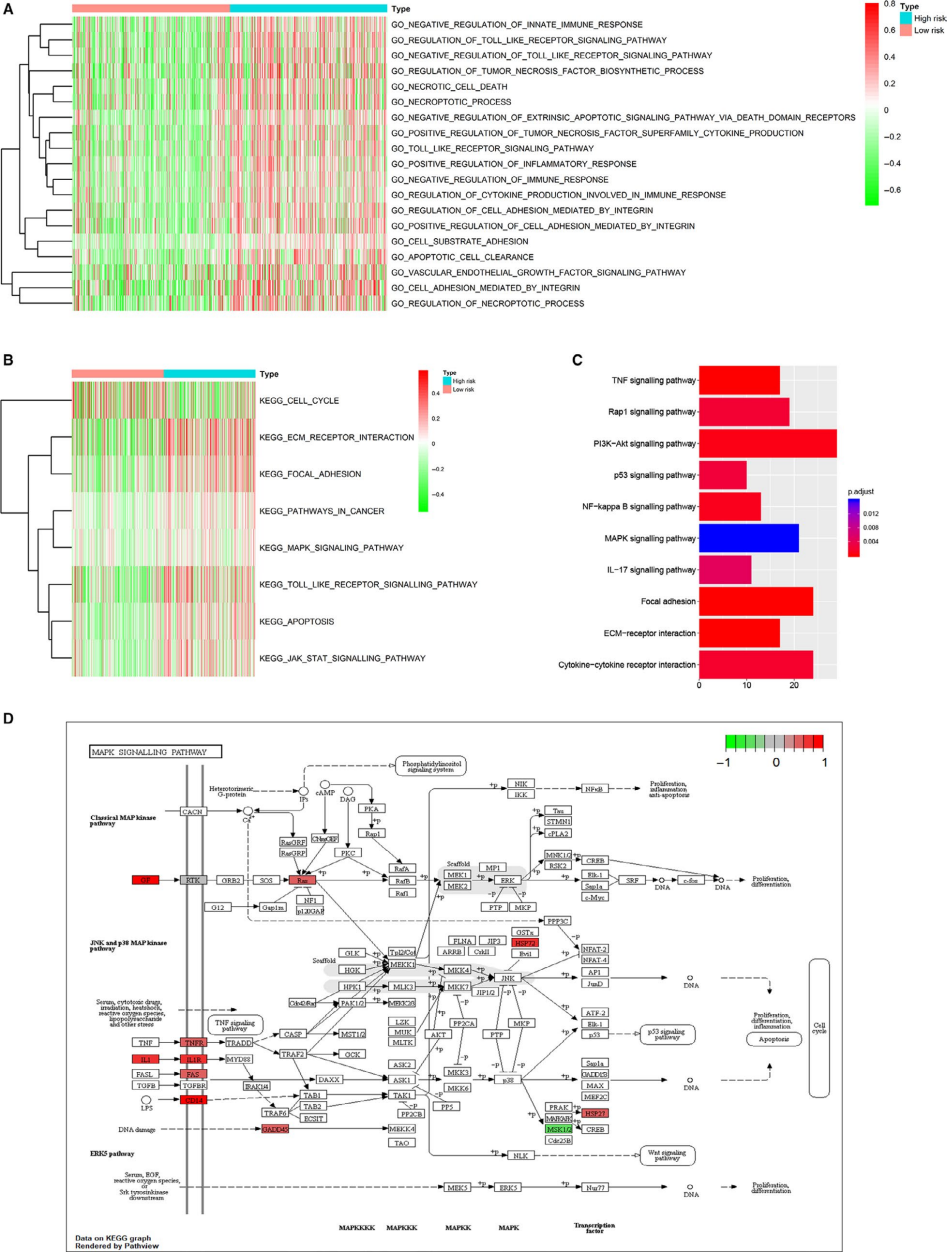
Functional roles of the risk signature. A, B, GSVA showed the biological processes (A) and KEGG pathways (B) associated with the risk signature. C, The risk signature‐related KEGG signalling pathway based on DEGs between low‐risk and high‐risk groups using R package “clusterProfiler”. D, MAPK signalling pathway map downloaded from the KEGG database was marked according to DEGs

GSVA was used to explore biological processes and KEGG pathways associated with the risk signature. As shown in Figure 7A, several biological processes relevant to apoptosis, necrosis, cell adhesion, immune and inflammatory response were enriched in the high‐risk group. These biological processes such as apoptosis, necrosis, immune and inflammatory response were closely related to autophagy.^19^, ^20^ For KEGG pathway, high‐risk group was positively correlated with focal adhesion, MAPK signalling pathway, Toll‐like receptor signalling pathway, apoptosis, ECM receptor interaction and so on (Figure 7B). To confirm the KEGG pathways associated with the risk signature, we screened out DEGs between low‐risk group and high‐risk group, and 558 genes were obtained (Table S3). R package “clusterProfiler” was performed on the DEGs. Consistent with the result in GSVA, KEGG pathways including focal adhesion, MAPK signalling pathway and ECM receptor interaction were also obtained (Figure 7C). Considering the close relationship between MAPK signalling pathway and autophagy, MAPK signalling pathway was downloaded from the KEGG database and marked according to DEGs such as HSP27, FAS and CD14 (Figure 7D). In this pathway map, a majority of red‐labelled genes were involved in triggering MAPK signalling pathway that can induce cell proliferation, differentiation, inflammation, cell cycle and apoptosis. In brief, these results revealed that the risk signature was correlated with apoptosis, necrosis, immunity and inflammation response and MAPK signalling pathway.

## 4 DISCUSSION

GBM is a refractory central nervous system tumour without effective treatment strategy. Emerging evidence has indicated that autophagy plays essential roles in GBM and serves as a therapeutic target. The level of autophagy in GBM is declined,^21^ and TMZ can promote autophagy in malignant glioma cells rather than apoptosis.^22^, ^23^ Furthermore, combination of TMZ with bafilomycin A1, an autophagy inhibitor, enhances the chemotherapy effect of TMZ for malignant gliomas.^22^ However, another study shows that TGF‐β2 induces autophagy to promote invasion of glioma.^24^ Despite the controversial roles of autophagy in GBM, these investigations suggest autophagy is a promising target for GBM treatment and needs further explorations.

In our study, we identified four autophagy‐related genes (DIRAS3, LGALS8, MAPK8 and STAM) which were associated with GBM survival. DIRAS family GTPase 3 (DIRAS3), also known as ARHI, is a member of RAS superfamily and locates on human chromosome 1p31. Its encoding protein shares 60% homology with RAS or Rap, but unlike other members, it has a long N‐terminal extension, low GTPase activity and constitutive GTP binding state in resting cells.^25^ DIRAS3 is involved in the occurrence and progression of a variety of cancers and considered as an anti‐oncogene. For example, DIRAS3 inhibits gastric cancer and epithelial ovarian cancer cells proliferation and promotes apoptosis by inducing autophagy.^26^, ^27^ Up‐regulating the expression of DIRAS3 can enhance the chemosensitivity in both breast cancer and ovarian cancer.^28^, ^29^ However, the functional roles of DIRAS3 in glioma were controversial. It has been reported that DIRAS3 is down‐regulated in glioma samples, and DIRAS3 overexpression inhibits the proliferation, migration and invasion of glioma cells.^30^ Instead, another study has shown that DIRAS3 is overexpressed in glioma and is positively associated with adverse outcome of glioma patients. In the meantime, overexpression of DIRAS3 promotes glioma cell proliferation and invasion via EGFR‐AKT signalling pathway.^31^ Our study was identical to the opinion that DIRAS3 was correlated with poor prognosis of GBM and was a risk factor for GBM survival. As a member of lectin family, LGALS8 (galectin‐8) plays key roles in various cellular processes, such as autophagy, cytoskeletal rearrangement, immunity and inflammation,^32^, ^33^, ^34^, ^35^ as well as tumour progression.^36^ LGALS8 can recognize lysosome damage and promote autophagy through inhibiting mTOR activity.^37^ Remarkably, LGALS8 may serve as prognostic biomarkers in cancers. The expression level of LGALS8 is associated with recurrence of gastric cancer, urothelial carcinoma of the bladder and clear cell renal cell carcinoma.^38^, ^39^, ^40^ In glioma, LGALS8 enhances the capacities of proliferation and migration and inhibits apoptosis of GBM cells.^41^ Consistent with our results, another research shows that GBM patients with higher expression level of LGALS8 have poorer prognosis.^42^ Mitogen‐activated protein kinase 8 (MAPK8), also known as JNK1, belongs to JNK kinase family that consists of three members: JNK1, JNK2 and JNK3.^43^ JNK directly binds and promotes phosphorylation of several transcription factors, such as NFAT, c‐Jun and JunB, and regulates multiple biological processes, including immune cell differentiation, inflammation, cancer progression and especially programmed cell death.^43^, ^44^, ^45^ JNK can trigger apoptosis, promote necroptosis, involve in non‐canonical pyroptotic signalling and induce autophagy by different molecular mechanisms.^46^ It has been reported that JNK1 affects chemosensitivity of colon cancer cells by regulating autophagy.^47^ In glioma cells, JNK1/c‐JUN signalling pathway enhances the transcription of CHAC1 which induces cell death and serves as a target of TMZ.^48^ Conformed to a DNA microarray data,^49^ our study also suggested that MAPK8 was a protective factor for GBM patients' survival. The fourth gene in our study is signal transducing adaptor molecule (STAM), which is a kind of phosphorylated tyrosine protein with distinctive structures containing a Src homology 3 (SH3) domain and an immunoreceptor tyrosine‐based activation motif (ITAM).^50^ STAM can be stimulated by various cytokines and growth factors, such as IL‐4, IL‐2, GM‐CSF and PDGF, and be involved in DNA synthesis, c‐myc induction, T cell development and neural cell survival.^50^, ^51^, ^52^ STAM is reported to be overexpressed in locally advanced cervical cancer.^53^ And another report shows that STAM may be responsible for regulating proliferation of gastric cancer.^54^ The research on STAM in GBM has not been reported yet, and it needs to be further explored.

Previously, there was a report about an autophagy‐related gene signature in glioma.^55^ According to the PCA and GSEA results that autophagy‐related genes were differentially distributed between GBM and LGG and preferred to enrich in GBM, we purposefully developed a prognostic signature with the four autophagy‐related genes in GBM. The results showing in ROC curves that the AUCs of the risk signature for 1‐, 3‐ and 5‐year survival prediction were larger than those of IDH1, MGMT promoter and CIMP status indicated the risk signature was more accurate for predicting the survival of GBM patients. In molecular function, we found the risk signature was correlated with apoptosis, necrosis, immunity and inflammatory response that were closely related to autophagy. Correspondingly, KEGG pathway analysis suggested that the risk signature was involved in MAPK signalling pathway. MAPK signalling pathway is essential for cell proliferation, differentiation and programmed cell death and regulates the balance of apoptosis and autophagy.^56^, ^57^ These results indicated the risk signature might serve as a therapeutic target for GBM. Another innovation in our study was that a nomogram that combined the risk signature and clinicopathological factors (IDH1 status, age and experience of radiotherapy or chemotherapy) was established for predicting 1‐, 3‐ and 5‐year survival rate of GBM patients. ROC curves and calibration plots validated an efficient performance of the nomogram. However, there were some deficiencies in our study. Firstly, the data came from several databases with limited sample size. Secondly, the prognostic factors in the nomogram did not contain the extent of GBM surgical resection which is a cardinal factor associated with GBM prognosis.^58^ Therefore, more samples with detailed clinical information will be collected to make up for these shortcomings and assess the efficiency of the nomogram in future study.

In summary, our study identified four autophagy‐related genes that were associated with GBM survival. Based on the four genes, a risk signature was established and proved to be an independent prognostic factor for GBM patients. On molecular function, the risk signature was found to be correlated with apoptosis, necrosis, immunity and inflammation response and MAPK signalling pathway. Furthermore, we developed a nomogram integrating the risk signature with several clinicopathological factors (IDH1 status, age and experience of radiotherapy or chemotherapy), which was validated to perform better for predicting 1‐, 3‐ and 5‐year survival.

## CONFLICT OF INTEREST

The authors declare no conflict of interest.

## AUTHOR CONTRIBUTIONS

WY, LX and GG conceived the concept and implemented the scheme. WY, ZM and ZW wrote the original draft. ZM and XZ reviewed and edited the manuscript. All authors read and approved the final manuscript.

## Data Availability

The data that support the findings of this study are available from the additional supporting information.
