# Supplementary material for: A risk signature with four autophagy‐related genes for predicting survival of glioblastoma multiforme
Source: J Cell Mol Med. 2020 Feb 17;24(7):3807–21. doi: 10.1111/jcmm.14938 (PMC7171404; doi:10.1111/jcmm.14938)
Supplement: Supplementary file 1 [file JCMM-24-3807-s001.docx]

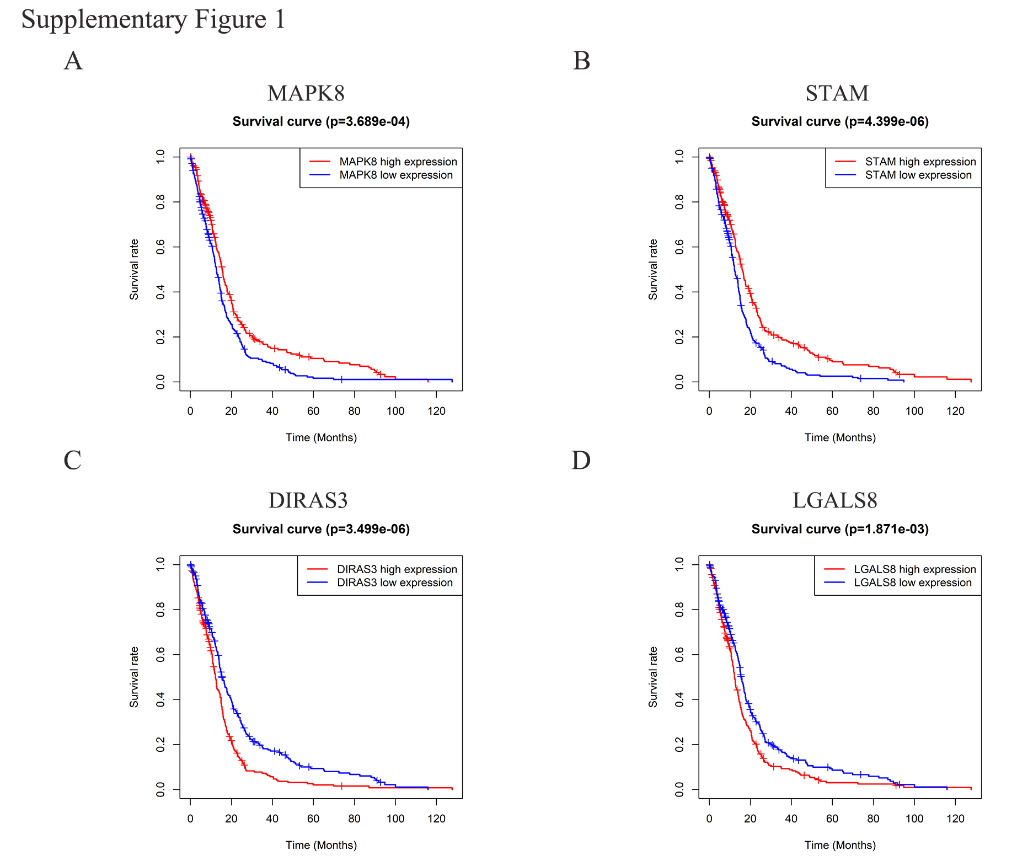


**Supplementary figure 1. The Kaplan-Meier survival curves showing the prognostic values of the four genes in GBM.**

(A-B) GBM patients with higher expression levels of MAPK8 or STAM had favorable prognosis (*P* < 0.05). (C-D) Higher expression levels of DIRAS3 or LGALS8 indicated poor prognosis in GBM (*P* < 0.05).
