# Supplementary material for: A risk signature with four autophagy‐related genes for predicting survival of glioblastoma multiforme
Source: J Cell Mol Med. 2020 Feb 17;24(7):3807–21. doi: 10.1111/jcmm.14938 (PMC7171404; doi:10.1111/jcmm.14938)
Supplement: Supplementary file 2 [file JCMM-24-3807-s002.docx]

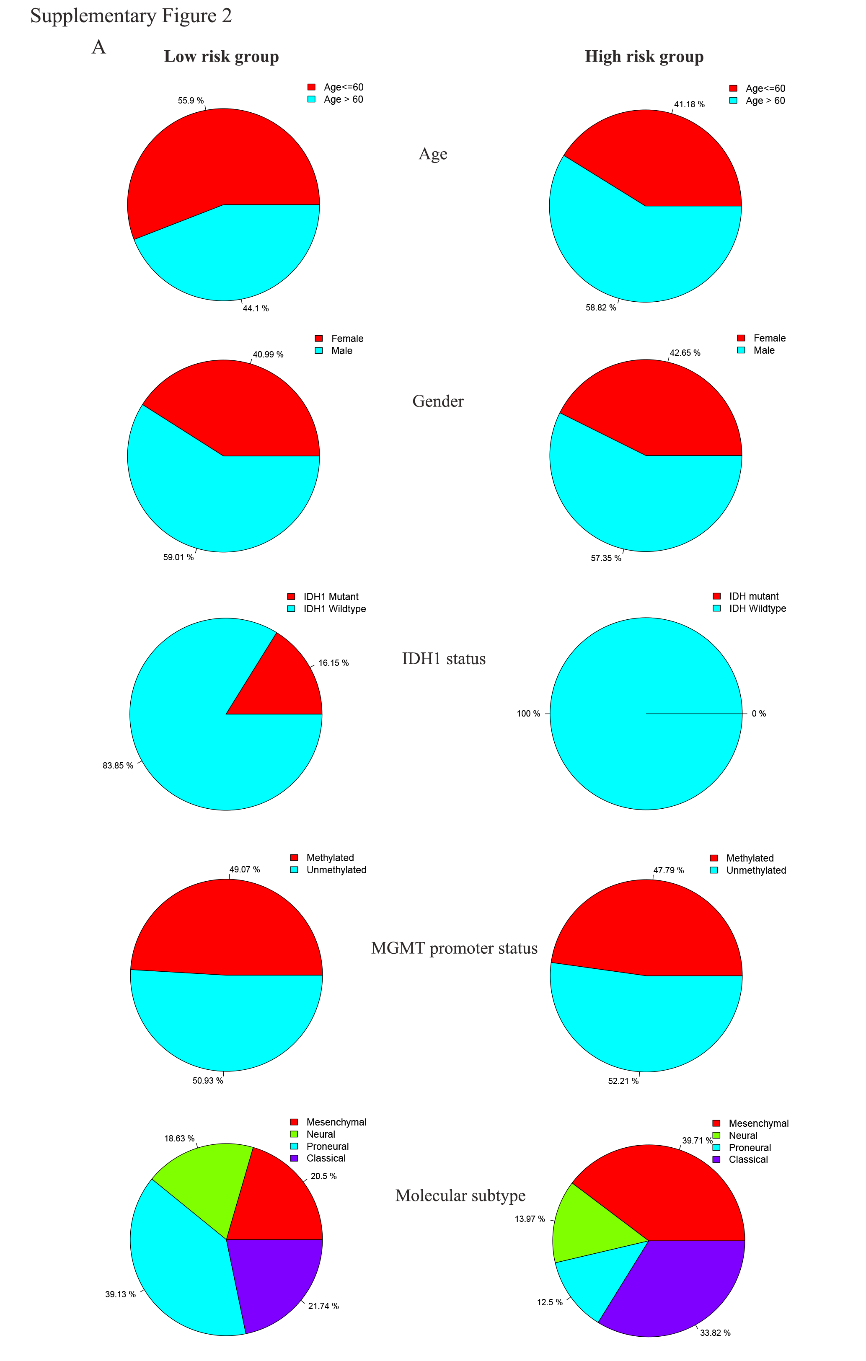


**Supplementary Figure 2. Pie charts showed the proportion of each factor (age, gender, IDH1 status, MGMT promoter status, molecular subtypes) in low-risk and high-risk groups.**
