# Supplementary material for: A risk signature with four autophagy‐related genes for predicting survival of glioblastoma multiforme
Source: J Cell Mol Med. 2020 Feb 17;24(7):3807–21. doi: 10.1111/jcmm.14938 (PMC7171404; doi:10.1111/jcmm.14938)
Supplement: Supplementary file 3 [file JCMM-24-3807-s003.docx]

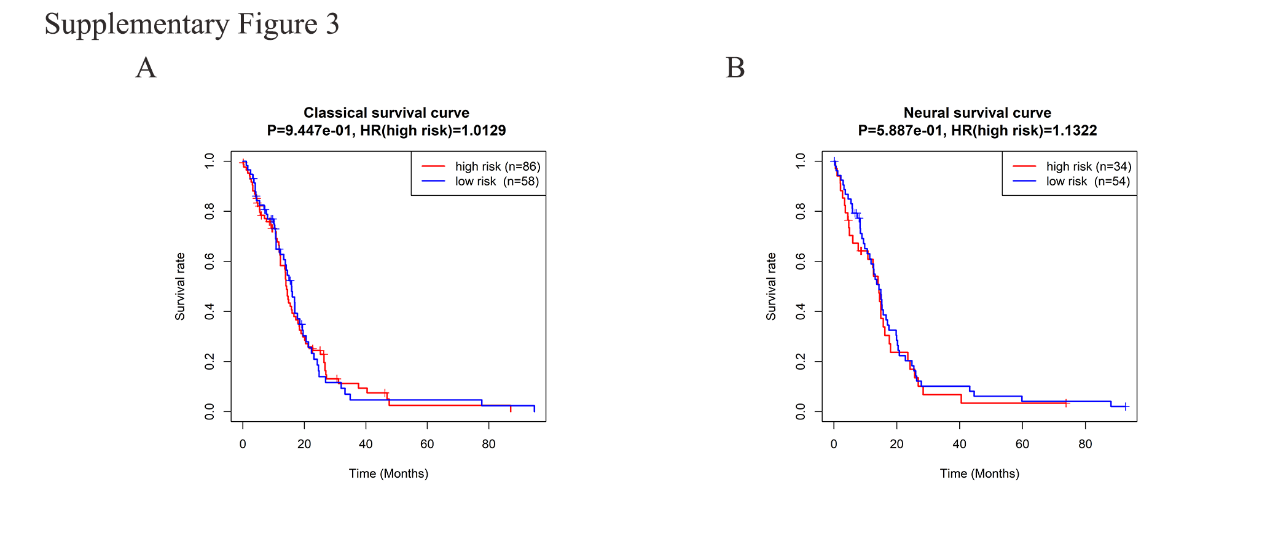


**Supplementary Figure 3. Prognostic values of the risk signature in the cohorts stratified by classical and neural subtypes (A-B)**
